# Epidemiological Study of Lung Cancer Incidence in Lebanon

**DOI:** 10.3390/medicina55060217

**Published:** 2019-05-28

**Authors:** Hamza A. Salhab, Mohamad Y. Fares, Hussein H. Khachfe, Hassan M. Khachfe

**Affiliations:** 1 Faculty of Medicine, American University of Beirut, Beirut 1107, Lebanon; myf04@mail.aub.edu (M.Y.F.); hhk15@mail.aub.edu (H.H.K.); 2 Neuroscience Research Center, Faculty of Medical Sciences, Lebanese University, Beirut 6573, Lebanon; 3 School of Arts and Sciences, and the Lebanese Institute for Biomedical Research and Application (LIBRA), Lebanese International University (LIU), Beirut 1105, Lebanon; hassan.khachfe@liu.edu.lb

**Keywords:** lung cancer, oncology, epidemiology, Lebanon

## Abstract

*Background and Objectives:* Lung cancer (LC) is the most common cancer in the world. Developing countries in the Middle East and North Africa (MENA) region, including Lebanon, have witnessed a great increase in the incidence rates of this disease. The aim of our study is to investigate the incidence rates of lung cancer in Lebanon from 2005 to 2015 and to compare these rates to other countries from the MENA region and other regions of the world. *Material and Methods:* Lung cancer data for the years 2005–2015 were collected from the National Cancer Registry of Lebanon and stratified by gender and age group. Age-specific and age-standardized incidence rates were calculated and analyzed using joinpoint regression. Age-standardized incidence rates to the world population (ASR(w)) for other countries were obtained from two online databases. *Results:* Lung cancer ranked as the second most common cancer in Lebanon and accounted for 9.2% of all newly diagnosed cancers. Lung cancer ASR(w) showed a significantly increasing trend over the period studied for males and females. Lung cancer ASR(w) among males in Lebanon came second after Malta when compared to other MENA countries, but it was among the lowest when compared to non-MENA countries. For females, Lebanon ranked first when compared to other MENA countries but was among the lowest when compared to countries in other regions of the world. The lung cancer incidence rate increased with age in both sexes and 89.2% of patients were 50 years of age or older. *Conclusion:* Lebanon has the highest incidence of LC in females and the second highest for males in the MENA region. The lung cancer incidence rate is on the rise and older age groups are much more burdened by this disease than the young ones. Several risk factors, particularly smoking, play a role in increased LC incidence among the Lebanese population.

## 1. Introduction

Cancer is the second leading cause of death in the world after cardiovascular disease [1,2]. It is associated with the highest social and economic burden of all causes of morbidity, mainly due to its expensive diagnostic tests and treatment, as well as the years of life lost due to disability [3].

Lung cancer (LC) is the most common cancer in the world in terms of new cases and deaths, as it accounts for 1.8 million new cases (12.9% of total) and 1.6 million deaths every year [4]. Developing countries in the Middle East and North Africa (MENA) region and other parts of the world have witnessed a great increase in the incidence rates of this disease [5].

Lebanon, a developing country in the Middle East, has an estimated population of 5,988,000 as of 2016 [6]. The Lebanese Ministry of Public Health (MoPH) is believed to have the most accurate count of all cancer cases after its data collection was initiated in 2003 by its National Cancer Registry (NCR). The registry aims to maintain a cancer incidence reporting system and to provide a primary source of population-based cancer cases for medical and public health investigators. In its data collection, the NCR relies on two main channels: capture and recapture systems. The capture system relies on physicians’ routine reporting of cases, either directly from their clinics or indirectly through the MoPH Drug Dispensing Center. On the other hand, the recapture system depends on gathering information from histopathologic and hematologic laboratories. The registry stratifies cases according to age, sex, and primary site. It has been reported that the NCR covers more than 90% of cancer cases in Lebanon [7]. The most recently published data by the NCR is from 2005 to 2015.

The aim of our study is to analyze the 11-year incidence rates of lung cancer in Lebanon, compare them to the results of other countries of the MENA region and other areas, and discuss the possible risk factors of lung cancer in Lebanon.

## 2. Materials and Methods

Lung cancer data (C-33 and C-34) using International Classification of Diseases for Oncology (ICD-O)(10th edition) were drawn from published data provided by the NCR database [8]. Age-specific and age-standardized incidence rates were calculated. The age-specific incidence rate is the number of new cancer cases that occurred during a specific time period in a population of a specific age and sex group divided by the number of midyear population of that age and sex group. The age-standardized rates to the world population (ASR(w)) are the incidence rates that would have been observed in our studied populations had they had the same age composition as a reference population. Our reference population is Ferlay’s modified world population [9]. This standardization is important for the comparison of results with other countries of different populations and age structures.

The computed age-specific rates and ASR(w) were analyzed using joinpoint regression analysis. The joinpoint model provides the annual percent change (APC) of LC incidence over the years studied and the detailed information of trends. Our statistical analysis was done using Joinpoint 4.7.0.0 with a significance level of 0.05. Our results were then compared with age-standardized and age-specific incidence rates from countries of the MENA region and random countries from other regions, as published in two online databases—the Cancer Incidence in Five Continents CI5XI [10] and CI5Plus [11]. These two databases are the result of a collaboration between the International Agency for Research on Cancer (IARC) and the International Association of Cancer Registries (IACR). The IACR is primarily for population-based registries that conform to accepted working practices and standards to ensure the completeness of the statistical data gathered.

## 3. Results

### 3.1. Overview

Over the 11-year period (2005–2015), 10,459 LC cases were reported in Lebanon. Males accounted for 68.7% (7186 cases) of these cases, while females accounted for 31.3% (3273 cases) of them. The male-to-female ratio was 2.3 between 2005 and 2015. During this period, LC was the second most common cancer after breast cancer in Lebanon and accounted for 9.2% (950 cases per year) of all newly diagnosed cancers. For males, LC came after prostate cancer in number of cases (12.9% of all male cancer cases, with an average number of 653 new cases per year) during the studied period. For females, breast and colorectal cancers were the two most common cancers, respectively, followed by LC, with an average number of 297 new cases per year, accounting for 5.6% of all female cancer cases. In general, the majority of patients were 50 years of age or older (89.2%).

### 3.2. Incidence Rates in Lebanese Males

**Figure 1:**
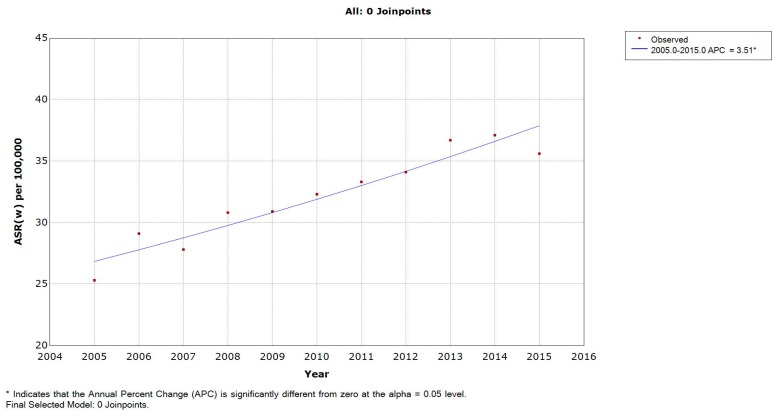
Age-standardized incidence rates (ASR(w)) (per 100,000) for lung cancer in males in Lebanon from 2005 to 2015.

**Figure 2:**
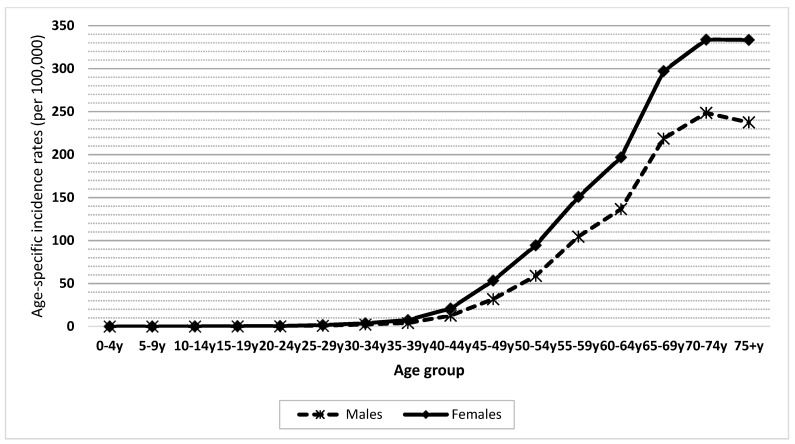
Gender and age-specific incidence rates (per 100,000 population) for lung cancer in Lebanon from 2005 to 2015.

The average LC ASR(w) over the period studied was 32.1 per 100,000. These rates increased from 25.3 to 35.6 per 100,000 (with a peak of 37.1 per 100,000 in 2014). The APC of LC age-specific rates significantly increased for the age groups 50–54, 65–69, 70–74, and 75+ years (Table 1). The APC of males’ ASR(w) was found to be 3.51% and significantly different from zero (Figure 1). The incidence rate of LC increased with age, reaching a maximum of 248.5 in males of the age group 70–74 years (Figure 2).

The LC ASR(w) among males in Lebanon (30.5 in 2005–2012) came second after Malta (35.2 in 2005–2012) when compared to other MENA countries. However, the LC ASR(w) among Lebanese males was among the lowest when compared to non-MENA countries such as Germany (50.2 in 2005–2012) and Japan (45.9 in 2005–2010) (Table 2).

### 3.3. Incidence Rates in Lebanese Females

**Figure 3:**
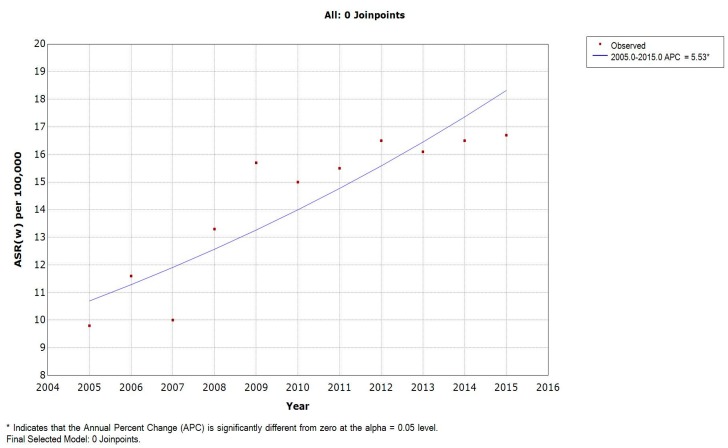
Age-standardized incidence rates (per 100,000) for lung cancer in females in Lebanon from 2005 to 2015.

The average LC ASR(w) over the period studied was 14.3 per 100,000. These rates increased from 9.8 to 16.7 per 100,000 (with a peak of 16.7 per 100,000 in 2015). The APC of LC age-specific rates significantly increased for the age groups 50–54, 55–59, 60–64, 65–69, and 70–74 years (Table 3). The APC of females’ ASR(w) was found to be 5.53% and significantly different from zero (Figure 3). The incidence rate of LC increased with age, reaching a maximum of 85.4 in the 75+ years age group (Figure 2).

The LC ASR(w) among females in Lebanon ranked first (13.4 in 2005–2012) when compared to other MENA countries but was among the lowest when compared to non-MENA countries such as Canada (31.9 in 2005–2012), Denmark (36.5 in 2005–2012), and others (Table 4).

## 4. Discussion

LC was the second most common cancer in males following prostate cancer and the third most common cancer in females following breast and colorectal cancers. During the studied period, the male-to-female LC incidence ratio was 2.3. Several studies have found that LC is a different disease in women then in men, with a different histological distribution [12]. In addition, studies suggest that women who have never smoked are more likely to develop LC than men who have never smoked [13]. However, whether men or women are more susceptible to the carcinogenic effects of tobacco smoke is still debatable [14]. A recent systematic review and meta-analysis by Yu et al. concluded that males have higher susceptibility for cigarette-attributable LC than females [15].This might explain the higher incidence of LC in Lebanese men, as cigarette and waterpipe consumption is more prevalent among them than in Lebanese females [16].

LC ASR(w) showed a generally significant increasing trend for both males (APC = 3.51%) and females (APC = 5.53%). While this can be explained by the increasing efficiency of the cancer registration system, it can alternatively suggest an increase in the burden and rate of the disease with time. The fact that Lebanon is a middle-income country further increases the burden because of the less-developed healthcare systems in poorer economies [17]. On the global level, lung cancer incidence rates had decreased dramatically by the end of the 20th century for males. On the contrary, LC rates in women increased since 1965 and showed a slight decline after 2000 [18]. When compared to other countries in the MENA region, Lebanon had one of the highest lung cancer incidence rates. For males, Malta ranked first with the highest LC ASR(w) in the MENA region, followed by Lebanon, whereas for females, the latter came first. This might be attributed to tobacco cigarette smoking. A direct response relationship has been reported between the number of cigarettes smoked and the risk of lung cancer [19,20,21]. This would explain in part the relatively high ASR(w) of lung cancer in Lebanon, as it has a high prevalence of active and passive smoking due to its weak tobacco control regulatory environment [22,23,24]. Moreover, the percentage of cigarette smokers in Lebanon (around 42.9% of male adults and 26.3% of female adults) is higher than that in the United States and Europe [25,26]. The concentration of tobacco-smoke-derived particle levels in indoor Lebanese public places was found to be among the highest three countries in a survey that included 32 countries [27].

ASR(w) for lung cancer in Lebanon for both males and females was among the lowest when compared to non-MENA regions. These results agree with previous studies that reported Central and Eastern Europe as well as Eastern Asia as having the highest LC incidence rates worldwide [4]. This might be attributed in part to the protective role that healthier Mediterranean dietary habits play against epithelial cancers [28], contrary to the fat- and sodium-rich Western diet that was estimated to be responsible for one-third of cancer deaths in Western countries [29]. Also, the limited healthcare funding in Lebanon and other low-middle-income MENA countries might hinder early cancer detection, thus underestimating the LC incidence rate [30,31].

Lung cancer is usually uncommon in people younger than 55 years, as it is partly a disease of aging [18,32]. This explains our results, as a significantly increasing trend in LC incidence was only found in 50+ years age groups, and 89% of LC patients in Lebanon were 50 years or older.

Other risk factors play a role in increasing the incidence of lung cancer in Lebanon. Relatively high air pollution due to diesel-fueled electric generators that are abundantly present in Lebanon as well as the high traffic load in different Lebanese areas are major risk factors [33,34]. Exposure to diesel exhaust has been associated with a 30%–50% increase in the relative risk for lung cancer [35,36].

Inherited variant alleles of the genes that encode glutathione-S-transferases (GSTM1 and GSTT1), proteins involved in metabolism of tobacco carcinogens (cytochrome P450-CYP1A1 gene), as well as other genes responsible for DNA damage repair (XPA, XPC, XPD, etc.) are associated with increased susceptibility to lung cancer [32,37]. The types of point mutations of these genes are consistent with the overall mutational spectra induced by tobacco carcinogens [38]. No significant data have been published concerning the expression of the abovementioned gene patterns in the Lebanese population. This highlights the need for more detailed genetic testing among people in Lebanon for assessing the risk of developing lung cancer. Also, genetics and epigenetics play a crucial role in targeted cancer therapies, which can be utilized in Lebanon should such studies become available.

Other risk factors include pre-existing inflammatory processes and lung diseases, particularly asthma and chronic bronchitis [39]. When compared to 23 other Eastern Mediterranean countries, chronic obstructive pulmonary disease (COPD) prevalence in Lebanon ranked third after Pakistan and Morocco [40]. Moreover, according to a recent Lebanese study including 2201 individuals, the prevalence of COPD was 9.7%. Among COPD patients, only 20.2% had been diagnosed and treated by a physician [41]. Also, asthma is a risk factor for COPD development and they can coexist in clinical settings [42]. Thus, the high prevalence of COPD and the probable coexistence of asthma among the Lebanese population contributes to the high LC incidence rates.

Moreover, recent studies have suggested a possible association of *Mycobacterium tuberculosis* and human papillomavirus (HPV) with increased risk of lung carcinoma [43,44]. This plays a role in the high LC incidence, as the tuberculosis population in Lebanon has been on the rise since 2007 [45]. Also, several studies have provided evidence pertaining to the high mortality rate among the population infected with human immunodeficiency virus (HIV) due to lung cancer [46,47]. It has been reported that this might be due to declining CD4 levels leading to increased LC rate [48], the increased risk of malignancy development caused by immunosuppression therapy [49], or simply because of the improved survival and aging of the HIV-positive population [47]. In Lebanon, the most recently published data show a low incidence of HIV (0.02 per 1000 population) [50]. However, underdetection of incidence cases of HIV may be a key factor behind the low incidence of HIV in Lebanon [51,52].

## 5. Conclusions

Analysis of data provided by the Lebanese National Cancer Registry showed that Lebanon has the highest incidence of LC in females and the second highest for males in the MENA region. The lung cancer incidence rate is on the rise and older age groups are much more burdened by this disease than the young ones. The high cigarette and waterpipe consumption among the Lebanese population plays a key role in addition to several other risk factors. Although several campaigns have been launched to increase awareness about the dangers of smoking, it remains prevalent in the Lebanese population, especially among adolescents and adults, with no sign of declining in the coming few years. Smoking cessation in public and closed places should be implemented, serious measures must be taken to limit the chaotic access to cigarettes and waterpipes, and more awareness campaigns should be launched targeting different age groups, particularly adolescents. Also, genetic and epigenetic studies should be done among the Lebanese population to better asses their effect in the development of lung cancer in Lebanon.

## Figures and Tables

**Table 1:** Trend analysis for lung cancer age-specific rate (per 100,000) in males by age group per year in Lebanon from 2005 to 2015.

Year	ASR(w)	Age-Specific Rate															
		0–4 y	5–9 y	10–14 y	15–19 y	20–24 y	25–29 y	30–34 y	35–39 y	40–44 y	45–49 y	50–54 y	55–59 y	60–64 y	65–69 y	70–74 y	75+ y
2005	25.3	0.6	0	0	0.5	1	0.7	2.8	4.8	14.1	35.5	38.4	69.6	115	162.4	147.1	222
2006	29.1	0	0	0	0.5	1	0.6	2	3.2	11.4	24	53.4	113.4	145.4	179.6	191.9	212
2007	27.8	0.7	0	0	0.5	0.5	0.6	2.8	1.7	9.3	29.6	48	106.3	124.7	189.3	197.3	188
2008	30.8	0	0	0	0	0	2.4	3.2	9.7	21.5	28.5	63.7	98.2	116.3	199.3	259.3	197
2009	30.9	0	0	0	0	0.5	1.8	1.9	1.6	15.3	29.2	38.8	112.8	140.4	217.7	227.4	235
2010	32.3	0	0	0	0	0	0	3.1	2.3	15.1	35.6	55.3	90.8	140.8	220.3	282.3	234
2011	33.3	0	0	0	0	0	1.1	0.1	9.2	9.3	41.8	60.5	122.2	113.3	246.2	280.2	209
2012	34.1	0	0	0	0	0.5	1.1	1.8	3	7.3	29.9	79.6	87.8	142.9	241.9	270.5	274
2013	36.7	0	0	0	0.4	0	0	1.6	2	7.3	30.8	70.9	134	172	253.1	295	247
2014	37.1	0.3	1.2	1	0.4	0.8	1.8	6	4.1	14	36.4	87.3	115.9	148.7	227.1	260.7	297
2015	35.6	0	0	0	0.3	0	0.4	1.3	4	14.1	28	52.2	99.8	142.9	268.9	321.4	297
APC	3.51 *	-	-	-	-	-	-	-	−0.03	−2.3	0.96	5.19 *	2.24	2.15	4.5 *	6.22 *	3.86 *

* Annual percent change (APC) significantly different from zero.

**Table 2:** Annual incidence rate (per 100,000) of lung cancer in males of different Middle East and North Africa (MENA) and non-MENA countries * (excl. Nunavut, Quebec, and Yukon).

	Country	Years	ASR(w)	Annual Incidence per 100,000 by Age Group: Males															
				0–4 y	5–9 y	10–14 y	15–19 y	20–24 y	25–29 y	30–34 y	35–39 y	40–44 y	45–49 y	50–54 y	55–59 y	60–64 y	65–69 y	70–74 y	75+ y
**MENA Countries**	Algeria (setif)	2008–2011	19.8	-	-	-	0.9	0.3	0.6	3.9	9.5	5.1	19.1	42	50.7	116.7	130.7	142.3	93.4
	Algeria (Batna)	2008–2012	11.8	-	-	-	-	-	0.4	0.5	1.1	5.8	8	20.9	39.5	69.2	76.5	88.2	70
	Bahrain	2005–2012	19.5	0.4	0	0	0	0	0	0.6	1.5	0.8	7.7	9.6	31	67.2	118	195	314.3
	Egypt (Damietta)	2009–2012	10	0	0	0	0.4	0	0.5	0.6	1.2	6.3	10.7	17	41.1	52.2	35	78.6	77.7
	Iran (Golestan)	2008–2011	15.1	-	-	-	-	0.5	0.5	1	2.5	8.6	10.5	32.1	44	73.5	75	137.3	126.1
	Jordan	2008–2012	18.1	0.1	-	-	0.1	0.3	0.3	0.5	2.1	8.9	21.2	38.1	46.8	74.5	118.1	155.6	133.6
	Kuwait	2005–2012	13.1	0.2	0	0	0	0	0	0.7	1.6	1.9	6.6	13.7	26.6	53.1	102.1	138.7	135.5
	**Lebanon**	**2005–2012**	**30.5**	**0.2**	**0**	**0**	**0.3**	**0.2**	**0.3**	**1.1**	**2.8**	**5.3**	**12**	**24.8**	**40**	**72.3**	**93.6**	**133.7**	**135.8**
	Malta	2005–2012	35.2	-	-	-	-	-	1.6	0.8	0.9	11.4	11.2	32.6	74.4	143.9	217.2	343.3	503.4
	Qatar	2008–2012	16.7	-	-	-	-	-	-	-	-	6.6	20	35.2	21.4	70.2	105	146.3	181.4
	Saudi Arabia	2008–2012	5.8	-	-	0.1	-	0.3	0.2	0.4	0.5	2.2	4.1	6	12.5	23.1	45.1	52.8	59.1
**Non-MENA Countries**	Turkey	2005–2012	74.9	0.1	-	0.1	0.2	0.4	0.5	1.9	7.1	26.5	71.3	143	247	371.2	490.8	577.6	455.2
	Cyprus	2005–2012	31	-	-	-	0.4	0.4	0.4	2.6	1.9	8.6	18.8	40.2	76.8	142.7	218.7	288.7	293
	Canada *	2005–2012	39.6	0.2	0.1	0	0.2	0.2	0.5	0.9	2	6.2	17.6	42.5	88	162.4	276.7	387.4	485
	Brazil, Goiania	2005–2012	23.5	-	-	-	0.2	0.2	1.2	0.5	2.1	6.3	12.5	30.7	51.9	108.2	165.8	238.7	216.3
	Thailand	2005–2012	28.7	0.1	-	-	0.1	0.6	1.7	2.9	6.4	11	19.4	45.9	71.8	113.2	193.6	273.5	256.5
	Denmark	2005–2012	43.4	-	-	0.1	0.1	0.3	0.2	1	3.2	8.1	21.1	51.9	111.3	187.2	288.3	413.1	481
	Germany	2005–2012	50.2	-	-	-	0.2	0.6	0.5	0.4	3.1	10.7	31.3	92	161	239.2	340.8	395.2	429.5
	Switzerland	2005–2012	38.8	-	-	0.2	0.2	0.2	0.8	2.1	1.6	9.1	24.1	55.9	108.7	180.1	258.5	335.8	374.9
	Japan	2005–2010	45.9	-	-	-	0.2	0.3	0.8	1.8	5	9.4	23.8	49.7	100.3	168.4	271.4	400.3	734.8
	Poland, Kielce	2005–2012	61	-	-	-	0.5	0.2	0.9	0.8	3.8	13.4	42.5	103.6	196.4	325	429	492.8	391
	Italy	2005–2010	46.7	0.1	-	-	0.2	0.4	0.8	0.8	2.7	7.5	21.9	48.8	109.3	209.4	302.6	426.4	592.6
	Costa Rica	2005–2011	9.4	0.1	-	0.1	-	0.5	0.4	0.4	0.6	1.7	5.2	9.1	18.1	33.8	66.6	83	132.8

**Table 3:** Trend analysis for lung cancer age-specific rate (per 100,000) in females by age group per year in Lebanon from 2005 to 2015.

Year	ASR(w)	Age-Specific Rates															
		0–4 y	5–9 y	10–14 y	15–19 y	20–24 y	25–29 y	30–34 y	35–39 y	40–44 y	45–49 y	50–54 y	55–59 y	60–64 y	65–69 y	70–74 y	75+ y
2005	9.8	0	0	0	0	0	1.2	1.3	5.4	6	13.8	20.3	30.1	55.2	46.4	33.9	78.7
2006	11.6	0	0	0	0	0	1.2	2.6	2	9.3	18.1	18.9	34.8	47.6	80	57.9	87.8
2007	10	0	0	0	1.1	0	0	0.7	6.1	9.3	8.4	27.3	39.1	37.9	35.7	74.5	69.2
2008	13.3	0	0	0	0	0.5	0.5	0	2.9	14	23.4	27.4	35.1	61.8	72	85.4	86.8
2009	15.7	0	0	0	0	0	2.2	1.7	3.5	10.3	29.5	29.3	52.4	59.3	76.6	108	121.1
2010	15	0	0	0	0.5	0	0.5	2.7	2.8	5.9	17.2	42	40.2	65.2	81.1	110.8	111.1
2011	15.5	0	0	0	0	0	0	0	2	9.2	24.3	39	53.2	71	104.5	95.1	72.9
2012	16.5	0	0	0	0	0	0.5	1.1	6	9	37.4	56.9	46.2	68.4	95.2	70.6	77.3
2013	16.1	0	0	0	0	0	0.9	1.9	1.8	3.7	25.3	45.6	53.9	61.6	91.7	103.3	112.2
2014	16.5	0	0.3	0.7	0	1.9	1.2	2.1	3.7	6.7	26	40.5	58.2	73.1	88.7	91.9	99.7
2015	16.7	0	0	0	0.4	0.4	0	0.8	1.6	8.5	14.8	44.5	66.4	62.6	92	108.3	139.3
APC	5.53 *	-	-	-	-	-	-	-	−5.64	−2.98	5.46	9.78 *	7.10 *	3.88 *	7.09 *	7.67 *	3.77

* APC significantly different from zero.

**Table 4:** Annual incidence rate of lung cancer in females of different MENA and non-MENA countries * (excl. Nunavut, Quebec, and Yukon).

	Country	Year	ASR(w)	Annual Incidence per 100,000 by Age Group: Females															
				0–4 y	5–9 y	10–14 y	15–19 y	20–24 y	25–29 y	30–34 y	35–39 y	40–44 y	45–49 y	50–54 y	55–59 y	60–64 y	65–69 y	70–74 y	75+ y
**MENA Countries**	Algeria (setif)	2008–2011	4.6	-	-	-	0.3	-	-	1.1	3.3	2.9	5.3	9.9	10.7	25.7	30.6	21.8	25.9
	Algeria (Batna)	2008–2012	1.9	-	-	-	-	-	-	-	-	0.6	2.1	4.5	6	11.1	12.1	12.1	12.2
	Bahrain	2005–2012	7.6	0	0	0	0	0	0.6	0.6	0	0.7	1.7	10.7	11.2	23.6	48.6	77.6	104.2
	Egypt (Damietta)	2009–2012	4.7	0.4	0	0	0	0.4	0.5	0	0.6	2	4.9	8.8	15.7	15.3	13.6	41.7	60.4
	Iran (Golestan)	2008–2011	6.5	0.3	-	-	0.3	0.5	1	1.7	2.1	4.1	3.6	12.6	17.9	17.8	49.3	37.9	61.8
	Jordan	2008–2012	3.8	0.1	-	-	-	0.1	0.1	0.8	1.1	1.7	5.8	7.6	8.9	14	24.6	30.1	29.6
	Kuwait	2005–2012	4	0	0.2	0	0	0.3	0	0	1.4	0.8	2.5	2.5	5	18.1	30.1	40.8	44.1
	**Lebanon**	**2005–2012**	**13.4**	**0.2**	**0.1**	**0**	**0.1**	**0.3**	**0.4**	**0.7**	**1.4**	**1.8**	**3.7**	**8.1**	**10.8**	**17.9**	**29.8**	**37.4**	**48.3**
	Malta	2005–2012	9.6	-	-	-	-	-	0.8	-	3.9	7.9	13.1	22.7	31.9	41.9	46.2	61.5	67.4
	Qatar	2008–2012	2.5	-	-	-	-	-	-	-	-	-	-	-	6.4	20.2	12.7	53.3	-
	Saudi Arabia	2008–2012	2.4	-	-	-	-	0.1	0.1	0.1	0.4	1	0.7	3.7	7	15.6	14	17	19.2
**Non-MENA Countries**	Turkey	2005–2012	2	-	0.1	0.1	0.1	0.3	0.2	0.9	2.5	6.3	13.1	21.2	27	39.2	55.2	60.3	61.7
	Cyprus	2005–2012	8.6	0.6	-	-	0.4	0.4	0.7	2.3	2.4	6.3	8.6	11.5	27	34.1	58.4	56.9	62.5
	Canada *	2005–2012	31.9	0.1	-	0	0.2	0.4	0.5	1	2.5	7.9	23.1	48.4	82.5	141.3	222.1	288.1	284.7
	Brazil, Goiania	2005–2012	12.6	-	-	-	0.2	0.2	0.4	1.6	1.2	7	12	23.3	32.1	44.1	94.9	96.6	114.3
	Thailand	2005–2012	14.4	-	-	0.1	0.1	0.4	0.8	2.7	3.3	6.7	12.1	22.4	39.2	58.1	96.3	121	122.2
	Denmark	2005–2012	36.5	-	-	-	0.2	0.2	0.4	1.4	3	9.9	28.6	70.5	108.7	169.8	234.7	310.4	272.7
	Germany	2005–2012	24.3	-	-	-	-	0.4	0.4	1.6	3.3	12.7	30.7	60	90.4	124.9	143.6	131.3	139.2
	Switzerland	2005–2012	20.2	0.2	0.2	-	0.2	0.5	1.4	1	3	7.3	20.8	41.5	65.3	97.3	127.8	148.2	127.1
	Japan	2005–2010	16.1	-	-	0.1	0.1	0	0.6	1.1	3.2	6.5	14.1	24.6	43.3	62	92.1	126.6	202.1
	Poland, Kielce	2005–2012	13.6	-	-	0.3	-	0.2	0.8	1.1	0.9	5.5	14.7	37.1	56.1	79.5	65.5	77.5	66.3
	Italy	2005–2010	14.1	0.1	-	-	0.2	0.1	0.9	1.5	2.6	5.4	15.9	32.8	41	62.8	73.7	96.9	128.9
	Costa Rica	2005–2011	4.5	0.1	-	-	-	-	0.4	0.4	1.1	1.3	3	5.9	10.9	17.9	31.1	36.1	48.7
